# Broadband Absorption Based on Thin Refractory Titanium Nitride Patterned Film Metasurface

**DOI:** 10.3390/nano11051092

**Published:** 2021-04-23

**Authors:** Dewang Huo, Xinyan Ma, Hang Su, Chao Wang, Hua Zhao

**Affiliations:** Institute of Modern Optics, Department of Physics, Harbin Institute of Technology, Harbin 150001, China; dwhuo@sina.com (D.H.); Haisuba@163.com (X.M.); suhanghit@126.com (H.S.); wangchao_hit@sina.com (C.W.)

**Keywords:** metasurface perfect absorber, thin film stacks, refractory material, titanium nitride

## Abstract

In this paper, a thin metasurface perfect absorber based on refractory titanium nitride (TiN) is proposed. The size parameter of the metasurface is investigated based on the finite difference time domain method and transfer matrix method. With only a 15-nm-thick TiN layer inside the silica/TiN/silica stacks standing on the TiN substrate, the near-perfect absorption throughout the visible regime is realized. The cross-talk between the upper and lower dielectric layers enables the broadening of the absorption peak. After patterning the thin film into a nanodisk array, the resonances from the nanodisk array emerge to broaden the high absorption bandwidth. As a result, the proposed metasurface achieves perfect absorption in the waveband from 400 to 2000 nm with an average absorption of 95% and polarization-insensitivity under the normal incidence. The proposed metasurface maintains average absorbance of 90% up to 50-degree oblique incidence for unpolarized light. Our work shows promising potential in the application of solar energy harvesting and other applications requiring refractory metasurfaces.

## 1. Introduction

Metasurface perfect absorbers (MPA) are of great significance for novel industries as one of the building blocks for various optical systems. The research of the MPA has drawn great attention since the first demonstration of a perfect absorber by Landy et al. to make use of the concept of metamaterials and show the promising potential to achieve strong absorption of light [1]. Regarding the working bandwidth, they can be categorized into narrowband absorbers and broadband absorbers. The narrowband absorbers can easily find applications as sensors [2], filters [3], display [4], etc., while, in the applications of solar energy harvesting [5], thermal emitters [6,7], radiative cooling [8,9], cloaking [10], etc., broadband absorbers are preferred. Noble metals, such as gold and silver, have been intensively researched in various absorber-related works [11,12]. In addition, high-index dielectrics [13,14] and novel materials (such as two-dimensional materials and water) [15,16] have also been integrated into the MPA designs. To date, the absorption range of MPAs covers from visible light to microwaves [1,5,17,18,19].

Although the MPAs provide impressive optical properties, they suffer from the photo-thermal effect, especially for broadband MPAs in the application of solar thermal energy harvesting [20]. Thus, many efforts have been made to design refractory MPAs based on the refractory metals such as tungsten (W), titanium (Ni) [21,22,23,24] and metal compounds such as titanium nitride (TiN) [25,26]. Titanium nitride, as one of the transition metal nitrides, has been presented as one of the promising candidates for noble metals and investigated in the visible and near-infrared regime to excite plasmonic resonances [25]. Various MPAs based on TiN achieve near-perfect absorption based on morphologies such as square, disk, cone or truncated-cone, tapered multilayer stacks, nanopillar, etc. [20,27,28,29,30,31,32] While some of the metasurface designs are difficult to fabricate including many layers or complicated morphology, others cannot achieve perfect absorption bandwidth.

In this work, we propose an MPA structure with TiN thin patterned layers embedded in silica standing on TiN substrate. The structure has four layers, and, for the unpatterned case, no nanofabrication steps are included beyond depositions of TiN and SiO${}_{2}$. Even for the patterned case, the structure can be completed via deposition and lithography. With the proposed structure, we realize near-perfect absorption in two wavebands, one is in visible regime from 400 to 800 nm with an average absorbance as high as 96.8% based on the simple SiO${}_{2}$/TiN/SiO${}_{2}$/TiN stacks, while the other is in the visible-to-near-infrared regime from 400 to 2000 nm with 95% absorption based on the stacks including patterned TiN thin layer.

## 2. Materials and Methods

**Figure 1:**
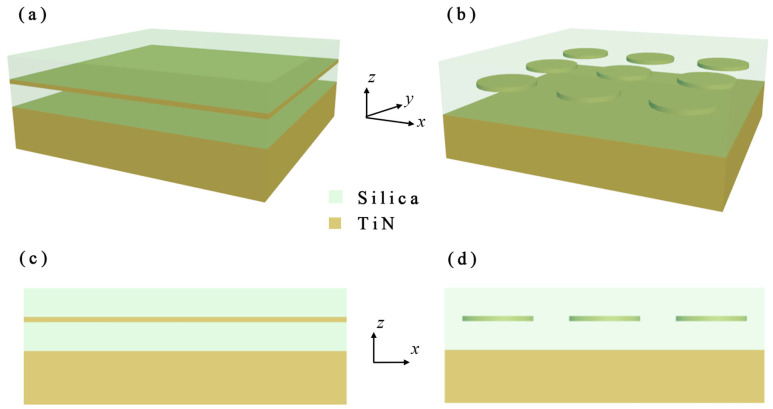
Schematics of proposed MPA consisting of SiO${}_{2}$/TiN/SiO${}_{2}$/TiN stacks: (**a**) perspective view; and (**c**) front-view. Schematics of proposed MPA consisting of SiO${}_{2}$/TiN-disk-array/SiO${}_{2}$/TiN stacks: (**b**) perspective view; and (**d**) front-view.

The proposed MPA is composed of four layers, as shown in the schematics depicted in Figure 1. The TiN thin film is embedded in the dielectric SiO${}_{2}$ layer. The thickness of the SiO${}_{2}$ super-layer above TiN film is denoted as $t_{1}$. The thickness of the SiO${}_{2}$ sub-layer underneath TiN film is $t_{2}$. The thickness of TiN film is denoted as $t_{3}$. The TiN film serves as a substrate with a thickness larger than the penetration depth of the electromagnetic wave in the studied waveband. The patterned nanodisks are arranged in a square lattice with a period. The analytical simulation of the thin layer stacks is based on the transfer matrix method, and the finite-difference time-domain method is adopted for the plasmonic modeling of the patterned TiN film stacks with the commercial software *FDTD Solutions* from *Lumerical Inc*. The light with *x*-polarization is assumed incident on the metasurface normally to the top surface of the structure along *z*-direction unless specified otherwise. The permittivity of the TiN and the dielectric are borrowed from Palik [33]. The absorbance A(ω) can be acquired from the reflectance R(ω) and transmittance T(ω), which are obtained in the simulation as

(1) A(ω)=1−R(ω)−T(ω)

The optimized absorption is achieved from the minimized reflection and transmission. Since TiN is used as the substrate and the thickness of the substrate is larger than the skin depth of electromagnetic wave in the studied waveband, the transmission is zero, and Equation (1) is simplified as

(2) A(ω)=1−R(ω)

Experimentally, the proposed structure with the layered stacks can be prepared through depositions of TiN and SiO${}_{2}$. Especially, the methods to fabricate a TiN film on a substrate include various methods, such as reactive magnetron sputtering [25,34,35], nitridation of TiO${}_{2}$ [36,37], chemical vapor deposition [38] and the atomic layer deposition (ALD) technique [39,40]. Then, the patterned thin film morphology can be prepared based on the photolithography and deposition techniques as described above.

## 3. Results and Discussion

### 3.1. Thin Film Stacks of TiN and Silica

**Figure 2:**
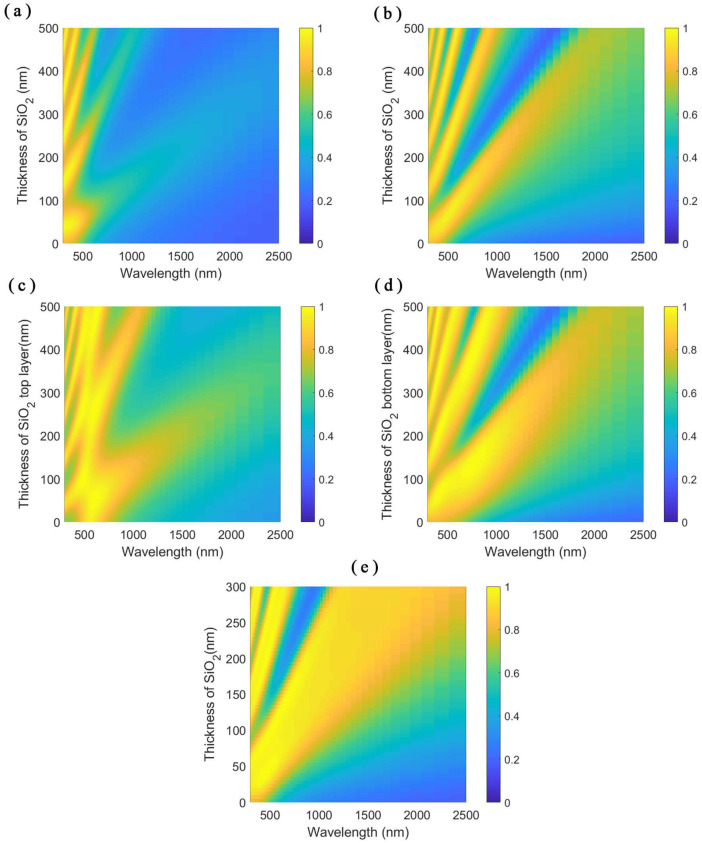
Absorption spectra of: (**a**) the SiO${}_{2}$/TiN stacks; and (**b**) the TiN/SiO${}_{2}$/TiN stacks with respect to the varying thickness of SiO${}_{2}$ layer. Absorption spectra of the SiO${}_{2}$/TiN/SiO${}_{2}$/TiN stacks with respect to: (**c**) the varying thickness of SiO${}_{2}$ layer on the top; (**d**) the varying thickness of the SiO${}_{2}$ layer underneath; and (**e**) the varying thickness of both SiO${}_{2}$ layers in the SiO${}_{2}$/TiN/SiO${}_{2}$/TiN four layer stacks. The thickness of the thin TiN layer is 15 nm.

At first, we consider light incident from the air onto the SiO${}_{2}$ film deposited on the TiN substrate. The reflection from the stacks can be calculated as the coherent sum of the partial waves reflected from the air/SiO${}_{2}$ interface and those reflected from the cavity after roundtrips. The absorption spectrum of SiO${}_{2}$/TiN stacks under the normal incidence is depicted in Figure 2a. Since the thickness of the TiN substrate is thick enough to prevent the penetration of incident electromagnetic waves, the TiN substrate can be viewed as semi-infinite. The SiO${}_{2}$ flat film is sandwiched between air and TiN. The two ends of the SiO${}_{2}$ form a cavity with a low-quality factor. The reflection dips (absorption peaks in the absorption spectra) result from the destructive interference of the reflection from the air/SiO${}_{2}$ interface and the partial reflection from the cavity after several roundtrips. As a result, the Fabry–Pérot (FP) cavity absorption pattern shows up in the absorption spectra plot, but it is not significant. The high absorption of the SiO${}_{2}$/TiN substrate at short wavelengths is from the absorption of the TiN substrate due to the absorptive dielectric property of TiN since the real part of the permittivity of TiN is positive and the imaginary part of the permittivity is large. As the wavelength of the incident electromagnetic wave gets longer, the real permittivity of TiN descends below zero to show metal-like property and the TiN substrate reflects most of the light.

Then, a thin TiN film is added upon the SiO${}_{2}$/TiN substrate structure. A cavity with a relative high-quality factor is formed. The absorption fringes in the absorption spectra in Figure 2b get a higher absorption peak-to-valley contrast, which indicates the high-quality factor of the cavity. Comparing the absorption plots of Figure 2a,b, the absorption spectra have the same absorption fringe order and the absorption in Figure 2b has higher values. The thin TiN layer on the top acts as an absorptive layer and reflective material in the TiN/SiO${}_{2}$ interface, which makes the structure a better cavity than the SiO${}_{2}$/TiN stacks to confine the electromagnetic waves in the cavity.

At last, we put another SiO${}_{2}$ layer on top of the TiN/SiO${}_{2}$/TiN stacks. The thin TiN in the middle separates the structure into two cavities. One is the low-quality factor cavity consisting of the stacks on top of the substrate, while the other is the high-quality factor cavity of the bottom SiO${}_{2}$ layer. The modes from the two cavities couple with each other. As the thickness of the SiO${}_{2}$ layer on the top increases, the cavity modes of the stack cavity couple with the modes in the bottom cavity as a result that absorption peaks of different modes have cross-talks, as shown in Figure 2c. When we fix the thickness of the SiO${}_{2}$ layer on the top and vary the thickness of the SiO${}_{2}$ layer underneath, the corresponding absorption spectra of the stacks are depicted in Figure 2d. The bandwidths of the absorption peaks broaden obviously compared to Figure 2b as a result of adding the SiO${}_{2}$ layer on top. In Figure 2c, we get the first cross-talk when the thicknesses of the top and bottom SiO${}_{2}$ layers are equal to each other. With the parameters in this case, a broadband absorber in the visible regime can be realized with an average absorbance up to 96.8% from 400 to 800 nm.

**Figure 3:**
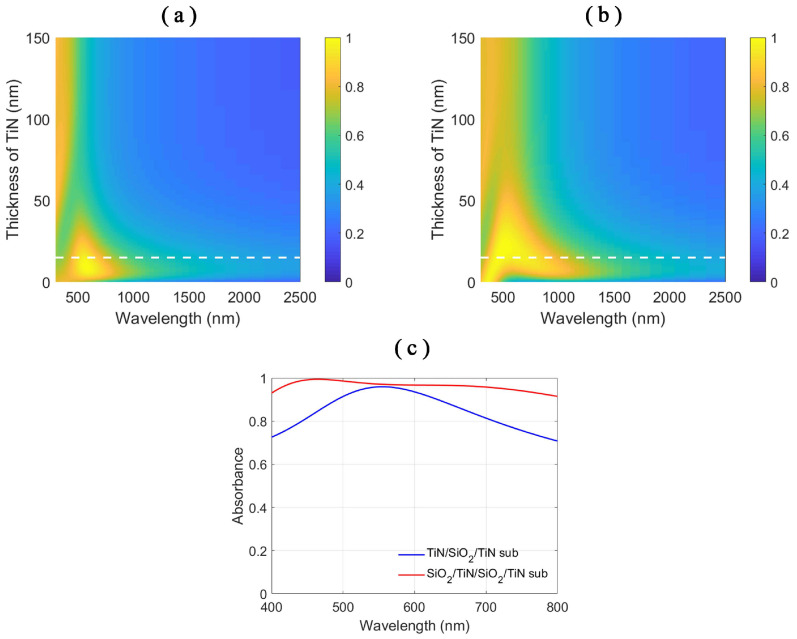
Absorption spectra of: (**a**) the TiN/SiO${}_{2}$/TiN stacks; and (**b**) the SiO${}_{2}$/TiN/SiO${}_{2}$/TiN stacks with respect to the varying thickness of the TiN layer. The thickness of the SiO${}_{2}$ layer is 85 nm. Color bar denotes the absorbance value of the stacks. (**c**) The absorbance plots of the stacks with size parameters chosen as the thickness of TiN layer on the top 15 nm, the thickness of the SiO${}_{2}$ layer 85 nm.

As the absorptive layer and the separation of the two cavities, the thin TiN layer also plays an important role in the absorption spectra. As the thickness of the thin TiN layer varies, the stacks with and without SiO${}_{2}$ superstrate show different responses to the incident electromagnetic waves, as shown in the absorption spectra in Figure 3a,b. When the thickness of the TiN layer is zero, the stacks equal to the SiO${}_{2}$/TiN substrate stacks with different SiO${}_{2}$ layer thicknesses of 85 and 170 nm, respectively. When the thickness of the TiN layer is large enough, little incident electromagnetic wave penetrates the TiN layer. The stacks in Figure 3a,b resemble air/TiN substrate and the 85-nm thick SiO${}_{2}$ layer standing on the TiN substrate, respectively. Varying the thickness of the TiN layer changes the energy into the SiO${}_{2}$ cavity under the TiN layer. The TiN layer also modulates the coupling strength of the resonances in stack cavity and bottom SiO${}_{2}$ cavity in SiO${}_{2}$/TiN/SiO${}_{2}$/TiN stacks, as shown in Figure 3b.

As the absorption performance of the SiO${}_{2}$/TiN/SiO${}_{2}$/TiN stacks is better than the TiN/SiO${}_{2}$/TiN stacks, we adopt the SiO${}_{2}$/TiN/SiO${}_{2}$/TiN stack structure for the further design. In Figure 2c,d, we choose the SiO${}_{2}$ layers with the same thickness to show the best coupling according to our calculation results. When the thicknesses of the two SiO${}_{2}$ layer vary simultaneously, the absorption spectra, as depicted in Figure 2e, show the same behavior of the cavity resonances with larger bandwidths of the absorption peaks compared to the cavity resonances in Figure 2a,b. The larger bandwidths come from the coupling of the cavity resonances in the stack cavity and bottom SiO${}_{2}$ cavity resulting in better impedance matching with the free-space [41]. The stacks can be designed to show perfect absorption in various wavelength ranges, single band or multiple bands. Due to the consideration of the realization of broadband absorption, we choose the first order of the cavity resonances which has a small slope, as shown in Figure 2e. We target the broadband absorption from wavelength 400 nm, so we choose the thicknesses of the SiO${}_{2}$ layers as 85 nm and the thickness of the TiN layer as 15 nm. Under this circumstance, the average absorption of the SiO${}_{2}$/TiN/SiO${}_{2}$/TiN stacks is 96.8% and the average absorption of the TiN/SiO${}_{2}$/TiN stacks is only 85.0% in the waveband from 400 to 800 nm, as shown in Figure 3c.

### 3.2. Thin Film Stacks of Patterned-TiN Film and Silica

The thin TiN film plays a crucial role in the broadband absorption of the film stacks. In addition to tailoring the thickness of the TiN layer, patterning the TiN layer into a nanodisk array will also change the absorption response of the stacks. The resonances in the nanodisk array may add other absorption peaks to the absorption spectra. Thus, we pattern the TiN thin film into the nanodisk array immersed in the SiO${}_{2}$ environment and study the effect of the size parameters of the array on the absorption performance. On the one hand, varying the size parameter of the nanodisk array changes the volume ratio of the TiN and the effective property of the layer to tailor the coupling of the resonances from the stack cavity and bottom SiO${}_{2}$ cavity. On the other hand, varying the size parameter of the TiN nanodisk array influences how the resonances in the nanodisk array respond in the absorption spectra.

**Figure 4:**
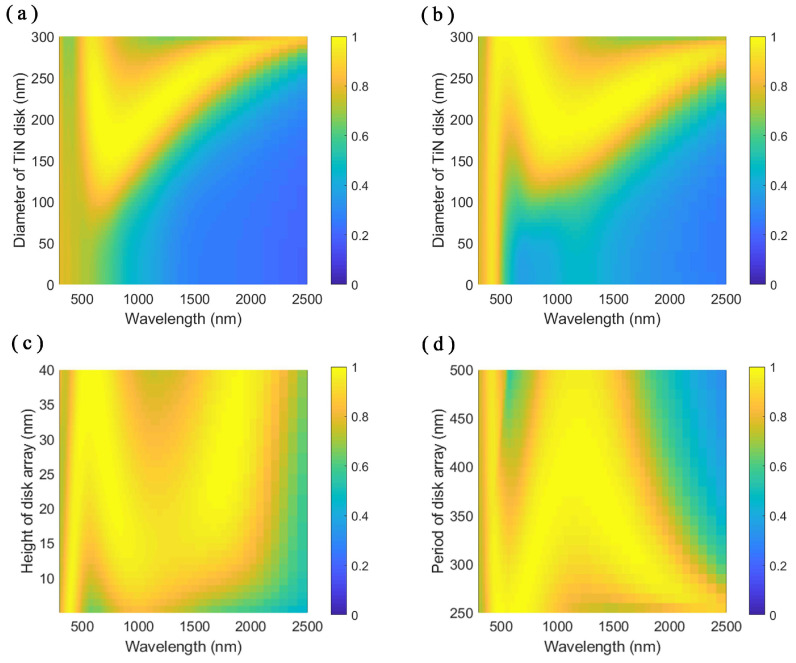
Absorption spectra with respect to the varying diameter of the nanodisk of: (**a**) patterned-TiN/SiO${}_{2}$/TiN stacks; and (**b**) SiO${}_{2}$/patterned-TiN/SiO${}_{2}$/TiN stacks. The height of the TiN nanodisk is 20 nm and the period of the nanodisk array is 300 nm. (**c**) Absorption spectra of the SiO${}_{2}$/patterned-TiN/SiO${}_{2}$/TiN stacks with respect to the varying height of the nanodisk. The diameter of the disk is 240 nm and the period of the array is 300 nm. (**d**) Absorption spectra of the SiO${}_{2}$/patterned-TiN/SiO${}_{2}$/TiN stacks with respect to the varying period of the nanodisk array. The diameter of the disk is 240 nm and the height of the disk is 20 nm.

The optical response of the structure depends on the size parameters of the structure including the size parameters of the nanodisk array. When the diameter of the TiN nanodisk is small compared to the period, as shown in Figure 4a,b, the patterned-TiN layer shows little influence on the incident electromagnetic wave due to a low filling factor of the TiN nanodisks. The structure resembles the structure of a SiO${}_{2}$ layer standing on the TiN substrate. Increasing the diameter of the TiN nanodisk, the patterned-TiN layer interacts more with the incident electromagnetic wave. As shown in the absorption spectra in Figure 4b, we can identify the left absorption peak as the cavity mode in the SiO${}_{2}$ layer. The other peaks in Figure 4a,b come from the influence of the TiN nanodisk array. As the diameter of the nanodisk increases gradually, the resonance mode splits into two modes and the absorption peaks separate each other. The three resonance peaks in the structure benefit the realization of the broadband perfect absorption. The superstrate SiO${}_{2}$ not only adds a peak to the absorption spectra but also widens the bandwidths of the two peaks from the effect of the nanodisk array compared to the no-SiO${}_{2}$ superstrate case.

We also simulated the influence of the height of the nanodisk and the period of the nanodisk array on the absorption spectra, as shown in Figure 4c,d, respectively. Similar to the variation of the diameter of the TiN nanodisk, the changes of the height and the period of the nanodisk array have influences on the absorption peaks induced by the patterned-TiN thin layer. As the height of the nanodisk increases, the first absorption peak redshifts slightly due to the increase of the whole structure thickness, and the other peaks separate from each other gradually. When the period of the nanodisk array gets larger, the two absorption peaks from the nanodisk array get close to each other. The first absorption peak from the cavity resonance changes little with the variation of transverse size parameters of the TiN nanodisk array since the refractive index of the TiN is comparable to that of the SiO${}_{2}$ in the short wavelength range.

**Figure 5:**
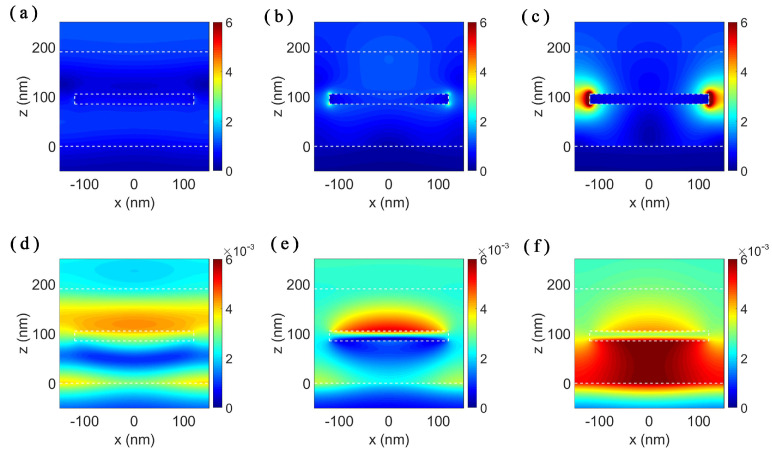
Electric (**a**–**c**) and magnetic (**d**–**f**) field distributions of the SiO${}_{2}$/patterned-TiN/SiO${}_{2}$/TiN structure in the *x-z* plane at wavelengths: (**a**,**d**) 467.4 nm; (**b**,**e**) 787.6 nm; and (**c**,**f**) 1779 nm. The size parameters of the stacks are as follows: thickness of the top and bottom SiO${}_{2}$ layer is 85 nm, the thickness of the patterned-TiN layer is 20 nm, the diameter of the TiN nanodisk is 240 nm and the period of the TiN nanodisk array is 300 nm. The white dashed lines outline the interfaces between different materials.

To see the modes of the structure, we calculate the electric and magnetic field distributions in the *x-z* cross-section of the MPA and plot them in Figure 5. We select the three absorption peaks located at 467.4, 787.6 and 1779 nm in the absorption spectra of the patterned-TiN nanodisk stacks. For the absorption peak at 467.4 nm, the electric field shows negligible enhancement and localization around the nanodisk due to the dielectric-like property of the TiN at this wavelength. The field patterns shown in Figure 5a,d resemble those in the TiN flat film stacks, indicating that the absorption peak at 467.4 nm comes from the cavity mode of the stacks. For the wavelengths of 787.6 and 1779 nm, TiN shows metal-like properties due to the negative real part of the permittivity. The strong electric field enhancement and localization emerge at the edges of the nanodisk, as shown in Figure 5b,c, which indicates the excitation of the surface plasmonic resonances in the metasurface structure. Thus, light is coupled into the structure and localized around the TiN nanodisk edges. The distributions of the magnetic field are intrinsically different. From the patterns of the magnetic field distributions, we can tell two different modes. For the first mode, the magnetic field is enhanced and localized along the top surface of the nanodisk as shown in Figure 5e. For the second mode, the magnetic field is enhanced and confined in the bottom SiO${}_{2}$ layer, as shown in Figure 5f. Thus, two different surface plasmon resonances result from the strong plasmon hybridization of the adjacent nanodisks [42].

**Figure 6:**
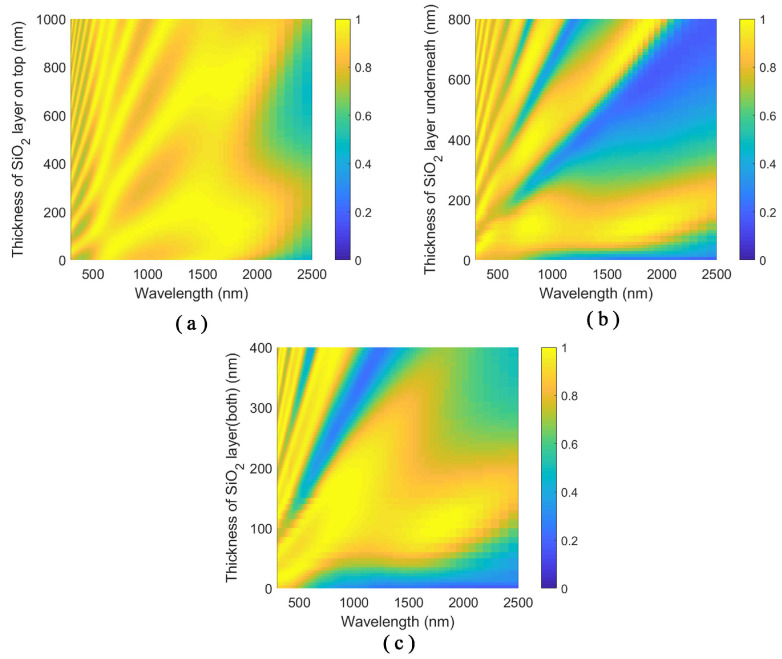
Absorption spectra of the SiO${}_{2}$/patterned-TiN/SiO${}_{2}$/TiN stacks with respect to: (**a**) varying the thickness of SiO${}_{2}$ layer on the top; (**b**) varying the thickness of the SiO${}_{2}$ layer underneath; and (**c**) varying the thickness of both SiO${}_{2}$ layers in the SiO${}_{2}$/patterned-TiN/SiO${}_{2}$/TiN four layer stacks. The thickness of the patterned-TiN layer is 20 nm.

The optical response of the structure also depends on the thickness of the top and bottom SiO${}_{2}$ layers. Varying the thickness of the surrounding SiO${}_{2}$ layer can tailor the absorption spectra of the structure. As shown in Figure 6a, the different order cavity modes couple with the modes from the patterned-TiN nanodisk array as the thickness of the top SiO${}_{2}$ layer varies. As a result, the broadband perfect absorption can be realized by the coupling of multiple absorption peaks. The mode coupling behavior is similar to the coupling in the SiO${}_{2}$/TiN/SiO${}_{2}$/TiN stacks. When the thickness of the bottom SiO${}_{2}$ layer varies, the absorption spectra from the structure exhibit a series of absorption peaks with an FP-like behavior, as depicted in Figure 6b. The variation of the thickness of the bottom SiO${}_{2}$ influences the interference of the FP mode of the SiO${}_{2}$ cavity and the gap surface plasmon resonances with effective refractive indices. As the thickness of the bottom SiO${}_{2}$ layer increases, higher-order gap surface plasmon resonances emerge. When the thicknesses of the top and bottom SiO${}_{2}$ layers vary simultaneously, the cavity mode and the gap surface plasmon modes shift with the same behavior. When the construction interference happens in the cavity, the absorption peaks show up, as shown in Figure 6. The absorption valleys result from the destructive interference of the modes in the cavity. The first-order modes are selected to serve the purpose of achieving broadband perfect absorption.

**Figure 7:**
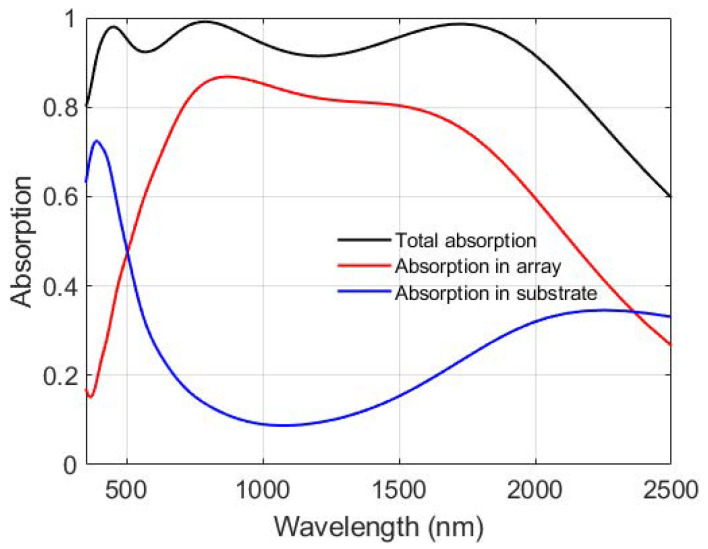
Absorption spectra of the different parts of the SiO${}_{2}$/patterned-TiN/SiO${}_{2}$/TiN stacks. The size parameters of the structure are the thickness of the patterned-TiN layer 20 nm, diameter of the disk 240 nm, period of the array 300 nm, and thickness of the SiO${}_{2}$ layers 85 nm.

The absorption of different parts of the patterned-TiN stacks is calculated, as depicted in Figure 7. The TiN substrate shows high absorption at short wavelengths corresponding to the first peak of the total absorption spectrum, which is attributed to the FP cavity mode in the stacks. The patterned TiN thin layer is responsible for the other two peaks in the total absorption spectrum which result from the surface plasmon modes of the nanodisk array. As seen in the magnetic field distribution in Figure 5f, the electromagnetic wave is confined in the bottom SiO${}_{2}$ layer. Thus, there is another peak in the absorption spectrum of the substrate at long wavelengths.

**Figure 8:**
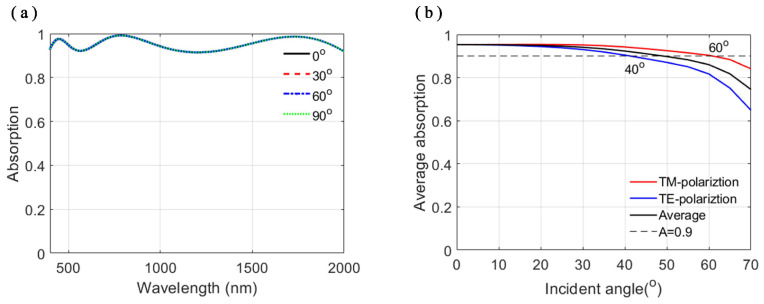
(**a**) Absorption spectra of the SiO${}_{2}$/patterned-TiN/SiO${}_{2}$/TiN stacks with respect to the varying polarization angle between the polarization direction and *x*-direction under normal incidence. (**b**) Average absorbance plot of the SiO${}_{2}$/patterned-TiN/SiO${}_{2}$/TiN stacks with respect to the varying incident angle for TM, TE and unpolarized light.

Due to the symmetry of the nanodisk and the square array arrangement, the patterned-TiN metasurface shows polarization-insensitivity under normal incidence, as shown in Figure 8a. As the incident angle increases, the polarization-insensitivity degrades to polarization-sensitivity. As shown in Figure 8b, the average absorption of the MPA decreases with the increase of the incident angle. For transverse-magnetic(TM) polarization, the proposed patterned-TiN nanodisk MPA remains as high as 90% absorbance under the oblique incidence up to 60${}^{\circ }$. For transverse-electric (TE) polarization, the proposed patterned-TiN nanodisk MPA remains as high as 90% absorbance under the oblique incidence up to 40${}^{\circ }$. Thus, for unpolarized light, the proposed MPA can remain an average absorbance of 90% under 50${}^{\circ }$ oblique incidence, as shown in Figure 8b.

## 4. Conclusions

We report a thin metasurface perfect absorber based on refractory titanium nitride. The absorbers based on SiO${}_{2}$/TiN/SiO${}_{2}$/TiN stacks and SiO${}_{2}$/patterned-TiN/SiO${}_{2}$/TiN stacks are studied, respectively. The thin-film stacks produce near-perfect absorption in the visible regime (400–800 nm) with an average absorbance of 96.8% due to the coupling of the FP modes. The stacks with patterned TiN layer achieve an average absorbance of 95% in the visible-to-near-infrared regime from 400 to 2000 nm, which is attributed to the cavity mode of the stack and the surface plasmon modes induced by the TiN nanodisk array. The proposed patterned-TiN MPA is polarization-insensitive under normal incidence due to the symmetry of the nanodisk and square lattice. The MPA remains an average absorbance more than 90% with a large receiving angle up to 60${}^{\circ }$ for TM polarization and 50${}^{\circ }$ for the unpolarized light. Our work may pave the way for solar-thermal application, and the strategy could be applied to other refractory materials.
